# A novel coumarin, (+)-3′-angeloxyloxy-4′-keto-3′,4′-dihydroseselin, isolated from *Bupleurummalconense* (*Chaihu*) inhibited NF-κB activity

**DOI:** 10.1186/s13020-016-0077-x

**Published:** 2016-02-13

**Authors:** Huai-Xue Mu, Cheng-Yuan Lin, Lin-Fang Huang, Da-Jian Yang, Ai-Ping Lu, Quan-Bin Han, Zhao-Xiang Bian

**Affiliations:** School of Chinese Medicine, Hong Kong Baptist University, Kowloon Tong, Hong Kong, China; Institute of Medicinal Plant Development, Chinese Academy of Medical Sciences, Beijing, 100193 China; Chongqing Academy of Chinese Materia Medica, Chongqing, 400065 China

## Abstract

**Background:**

This study aims to identify the major anti-inflammatory components in the petroleum ether extract of *Bupleurummalconense* (*Chaihu*), by bioassay-guided fractionation, and to investigate the anti-inflammatory mechanisms of active components in lipopolysaccharide (LPS)-stimulated murine macrophage RAW-Blue cells.

**Methods:**

A QUANTI-Blue assay was used to guide fractionation of *B.malconense* root extract. The petroleum ether extract which exerted significant secreted embryonic alkaline phosphatase (SEAP) inhibition effect was purified by silica gel column chromatography and assisted with reverse phase HPLC. The major bioactive compound which significantly inhibited SEAP activity was obtained and its anti-inflammatory effects in LPS-induced RAW-Blue cells were measured by the overproduction of NO (Griess method), gene expression of *Il*-*1β*, *Tnf*-*α* and *iNos* (real-time PCR). In parallel, protein expressions of COX-2, iNOS and IκB-α were determined by western blot.

**Results:**

In bioassay-guided fractionation using LPS-stimulated mouse macrophage RAW-Blue cells, (+)-3′-angeloxyloxy-4′-keto-3′,4′-dihydroseselin (Pd-Ib) was identified by MS and NMR spectral analyses. Pd-Ib (5, 10, 20 μg/mL) suppressed the gene expression of *Il*-*1β* (*P* < 0.0001, *P* < 0.0001, *P* < 0.0001 for three respective concentrations), *Tnf*-*α* (*P* = 0.006, *P* = 0.001, *P* < 0.0001 for three respective concentrations) and *iNos* (*P* = 0.009, *P* < 0.0001, *P* < 0.0001 for three respective concentrations) in LPS-stimulated macrophages. The production of cyclooxygenase-2 (*P* = 0.019, *P* = 0.002, *P* < 0.0001), iNOS (*P* < 0.0001, *P* < 0.0001, *P* < 0.0001 for three respective concentrations) and NO (*P* < 0.0001, *P* < 0.0001, *P* < 0.0001 for three respective concentrations) significantly decreased when macrophages were treated with Pd-Ib (5, 10, 20 μg/mL) in the presence of LPS. Pd-Ib (5, 10, 20 μg/mL) suppressed the nuclear activation of NF-κB while it up-regulated the IκB-α level (*P* = 0.028, *P* = 0.013, *P* = 0.005 for three respective concentrations) in LPS-stimulated macrophages.

**Conclusions:**

Pd-Ib isolated from *B.malconense* suppressed LPS-induced inflammatory responses in macrophages by inhibiting NF-κB activity and reducing the expression of iNOS, COX-2 as well as pro-inflammatory cytokines.

**Electronic supplementary material:**

The online version of this article (doi:10.1186/s13020-016-0077-x) contains supplementary material, which is available to authorized users.

## Background

*Bupleurum* is used for treatment of inflammation-related diseases, such as autoimmune diseases, inflammatory bowel syndrome and cholecystitis [1–3]. Nearly 50 *Bupleurum* species have been extensively studied for their phytochemical characteristics [4]. In general, the components of *Bupleurum* include essential oil and saponins [5, 6]. Saikosaponins are commonly recognized as the main components responsible for the anti-inflammatory activity of the *Bupleurum* species [7–9]. However, *Bupleurum malconense,* a species endemic to China, appears to little be known its major bioactive components [10–12]. In a preliminary study of our group, the anti-inflammatory effect of *B. malconense* was evaluated in dextran sulfate sodium (DSS)-induced colitis mouse model. The petroleum ether extract of *B. malconense* exerted strong ameliorative effect on colon shortening and loss of the body weight (Additional file 1). As saikosaponins are polar, we believed that some compounds less polar than saikosaponins may be responsible for the anti-inflammatory properties of the petroleum ether (PE) extract of *B. malconense.*

This study aims to identify the anti-inflammatory components in the PE extract of *B. malconense* by QUANTI-Blue bioassay-guided fractionation, and to investigate the anti-inflammatory actions of these active components in LPS-stimulated murine macrophage RAW-Blue cells.

## Methods

### Chemicals

Lipopolysaccharides (LPS, L3129), 3-[4, 5-dimethylthiazol-2-yl]-2, 5-diphenyltetrazolium bromide (MTT), dimethyl sulfoxide (DMSO), Griess reagent, and all chemicals used were of HPLC grade from Sigma Chemical Co. (St. Louis, MO, USA). Primers for inducible nitric oxide synthase gene (*iNos*), interleukin 1 beta gene (*Il*-*1β*) and tumor necrosis factor alpha gene (*Tnf*-*α*), beta-actin gene (*β*-*Actin*) (Table 1), Trizol reagent, SYBR Green, Dulbecco’s modified Eagle’s medium (DMEM), ECL reagent, fetal bovine serum (FBS), penicillin and streptomycin were purchased from Life Technologies (Carlsbad, CA, USA). BCA protein assay kit was purchased by Thermo Fisher Scientific (Waltham, MA, USA). Nuclear factor-kappa B (NF-κB), I-kappa B alpha (IκB-α), cyclooxygenase-2 (COX-2) and iNOS rabbit antibodies were purchased from Cell Signaling Technology (Beverly, MA, USA). β-actin mouse antibody, anti-rabbit IgG and anti-mouse IgG were purchased from Santa Cruz Biotechnology (Santa Cruz, CA, USA). QUANTI-Blue medium and Alexa Fluor568^@^ anti-rabbit IgG were purchased from InvivoGen (San Diego, CA, USA). Fractions were monitored and combined by thin layer chromatography. Spots were made visible by heating silica gel plates that had been immersed in 5 % H_2_SO_4_ in ethanol (EtOH).

**Table 1:** Sequence of primers used in real-time PCR

Gene	Primer	Sequence (5′–3′)
*iNos*	Sense	CACCTTGGAGTTCACCCAGT
	Antisense	ACCACTCGTACTTGGGATGC
*Tnf*-*α*	Sense	CTGTGAAGGGAATGGGTGTT
	Antisense	GGTCACTGTCCCAGCATCTT
*Il*-*1β*	Sense	GCTGAAGGAGTTGCCAGAAA
	Antisense	GTGCAAGTGACTCAGGGTGA
*β*-*Actin*	Sense	GGTGAAGGTCGGTGGAACG
	Antisense	CTCGCTCCTGGAAGATGGTG

### Plant material

The dried roots of *B.malconense* were collected from Sichuan Province, China. The plant material was identified and authenticated by Dr. Linfang Huang based on sequences of the plastid psbA-trnH intergenic region [13, 14]. The sequences of plant material (ZSH1 and ZSH2) highly matched the sequence of *B. malconense* (GenBank accession: JN788921) (Additional file 2).

### Extraction and isolation

Air-dried pieces of *B.malconense* root (500 g) were extracted three times by percolation in PE (1 L). The supernatant, which was concentrated and collected, was the PE extract. The residue was extracted three times by reflux in 80 % EtOH at 60 °C. The solution was concentrated to yield an 80 % EtOH. Then, the residue was further extracted by reflux in water (1 L) for three times to obtain the aqueous extract. Meanwhile, the 80 % EtOH extract was dissolved in 200 mL of water in a separatory funnel, and then partitioned with ethyl acetate (EtOAc, 200 mL × 3). The upper and lower layers were collected to yield the EtOAc extract and EtOH extract, respectively. After freeze-drying (Labconoco, Kansas, MO, USA), extracts were weighted, and the related percent composition was calculated, with the following results: PE extract (0.8 g, 0.2 %), EtOAc extract (4.7 g, 0.9 %), EtOH extract (25.3 g, 5.1 %), aqueous extract (24.2 g, 4.8 %). All extracts were stored at −20 °C.

**Fig. 1:**
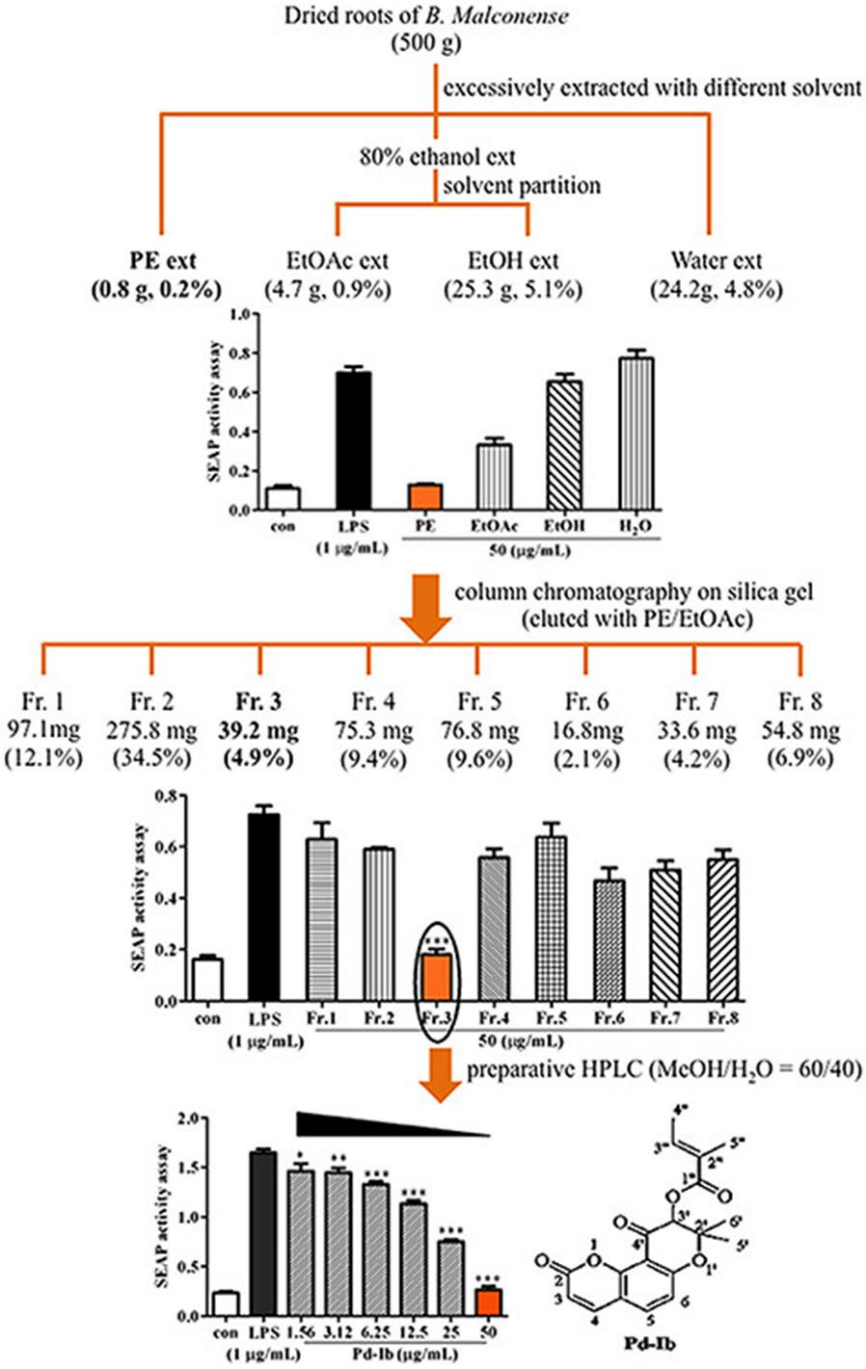
Flow chart of bioassay-guided isolation of the anti-inflammatory compound of *B. malconense*. The effect of test samples on SEAP activity in LPS-stimulated RAW-Blue cells. *P < 0.05, **P < 0.01 and ***P < 0.001 *vs.* LPS-alone group. *PE ext* petroleum ether extract, *EtOAc* ethyl acetate extract, *EtOH* ethanol extract, *H*_*2*_ *O* water extract, *Fr* fraction, *con* vehicle control group

The PE extract was subjected to silica gel column chromatography (300–400 mesh, Davisil, Germany) using PE/EtOAc with increasing polarity as eluent. Pd-Ib, which was obtained from fraction 3 (Fr. 3) with 2.5 % EtOAc in PE, was further purified by reverse phase HPLC (Waters system including a 2545 binary gradient module, a 2489 UV/Visible detector and a fraction collector III) on the semi-preparative column Preparative RP-C_18_ (Alltech Alltima-C_18_, 250 mm × 10 mm, 5 μm). The separation flow chart (together with bioassay) is shown in Fig. 1.

### Anti-inflammatory bioassay

RAW-Blue cells (5 × 10^4^/well) were cultured in 96-well plates for 24 h, and then treated with LPS (1 μg/mL) alone or together with test samples for 20 h. Secreted embryonic alkaline phosphatase (SEAP) activity in the medium was determined by QUANTI-Blue medium following the manufacturer’s instructions. Briefly, 100 μL of samples were added to 200 μL of QUANTI-Blue medium and incubated at 37 °C for 15–30 min. Absorbance was measured at 620 nm by a microplate reader (Benchmark plus Bio-Red, Hercules, CA, USA), and fold change in SEAP activity was calculated.

### Cell culture

RAW-Blue cells were derived from RAW264.7 murine macrophages. RAW-Blue cells were cultured in plastic dishes containing DMEM supplemented with 100 U/mL penicillin, 100 μg/mL streptomycin and 10 % FBS in an incubator (5 % CO_2_) at 37 °C. Cells were sub-cultured every 3 days at a dilution of 1:6.

### Cell viability assay

Cells (5 × 10^4^/well) were cultured in 96-well plates for 24 h, and then cultured with various concentrations of Pd-Ib (1.25, 2.5, 5, 10, 20, 40, 80, and 100 μg/mL) for 24 h. Then, 10 μL of 5 mg/mL MTT were added to each well, and the cells were cultured in the dark for 3 h. The medium was then discarded, and 100-μL portions of DMSO were added into each well. After 15 min incubation, the optical density at 570 nm was measured by a microplate reader to determine the cell viability.

### Western blot analysis

After RAW-Blue cells were treated with LPS (1 μg/mL) and various concentrations of Pd-Ib (5, 10, 20 μg/mL) for 24 h, total proteins were extracted by lysis buffer [150 mM NaCl, 1 mM EDTA, 1 % NP-40, 2 mM EGTA, 50 mM Tris (pH 7.4)] with phosphatase and protease inhibitors (Roche, Mannheim, Germany). Cell extracts were centrifuged by centrifugation (5424R, Eppendorf, Hamburg, Germany) at 17,000×*g* for 15 min at 4 °C. The amount of total protein concentration was quantified by the BCA protein assay kit (Thermo Fisher Scientific, Waltham, MA, USA). Then, 10–25 μg proteins were loaded and separated on sodium dodecylsulphate-polyacrylamide gel (10 % SDS-PAGE), which were further transferred onto polyvinylidene difluoride membranes. The membranes were incubated with various primary antibodies, namely, anti-COX-2, anti-iNOS, anti-IκB-α, and anti-β-actin (1:5000) in 5 % skim milk (wt/vol) in TBST overnight at 4 °C, after blocking with 5 % skim milk (wt/vol) in Tris-buffered saline-Tween [TBST; 150 mM NaCl, 50 mM Tris, 0.05 % Tween-20 (pH 7.4)] for 1 h. After washing with TBST for three times (10 min for each time), the membranes were blocked with secondary antibodies (1:2000) in 5 % skim milk (wt/vol) in TBST for 1 h, following with TBST washed (3 × 10 min). The amount of immunoreactive proteins was detected by enhanced chemiluminescence ECL reagent and X-ray film. The protein bands were quantified using the ImageJ Software (version 4.1.7, NIH, Bethesda, MD, USA) by their relative intensity compared with the control.

### Immunofluorescence staining

RAW-Blue cells were cultured (2 × 10^5^ cells/well) on glass coverslips and incubated for 24 h. Cells were pretreated with Pd-Ib (20 μg/mL) for 2 h and then with LPS (1 μg/mL) for 20 h. Subsequently, the coverslips were rinsed twice with phosphate buffer saline, and cells were fixed in 4 % paraformaldehyde in PBS at room temperature for 15 min. Cellular and nuclear membranes of the macrophages were permeabilized by treatment with 3 % Triton X-100 in PBS for 15 min. After being blocked with 3 % bovine serum albumin (BSA) in PBS for 1 h, the cells was incubated with primary antibodies in 3 % BSA/PBS (1:500 dilution) at 4 °C overnight. After washing with PBS, the cells were incubated with the Alexa Fluor568-conjugated secondary antibodies (1:500 dilution) in 3 % BSA/PBS at room temperature for 1 h, and finally washed again three time with PBS. Then, counter-staining of nuclei was performed with 4′,6-diamidino-2-phenylindole (DAPI; 1:1000 dilution) in 3 % BSA/PBS for 10 min. The cells were washed three times with PBS, and then anti-fade mounting medium was added. Samples were observed under a fluorescence microscope (DMI3000B, Leisa Microsystems, Wetzlar, Germany).

### Real-time quantitative polymerase chain reaction (qPCR) analysis

2 × 10^6^ cells/well RAW-Blue cells were seeded in 12-well plates. After 24 h, cells were pretreated with Pd-Ib at different concentrations (5–20 μg/mL) or untreated (control) for 2 h, following co-cultured with LPS (1 μg/mL) for 20 h. TRIzol reagent was used to extract the total RNA. Then, 2 μg of total RNA was reverse-transcribed to cDNA with RT master mix. Real-time qPCR was performed with the SYBR Green kit in a ViiA7 real-time PCR instrument (Life Technologies, Waltham, Massachusetts, USA). The comparative cycle of threshold (ΔΔC_T_) method of relative quantification was used to analyze the target gene expression. The accuracy of the amplicon was verified using melting curve analysis. Primer sequences of *Il*-*1β*, *Tnf*-*α*, *iNos* and *β*-*Actin* for mRNA analysis are listed in Table 1.

### Nitric oxide production determination

Nitric oxide (NO) production was indirectly assessed by measuring the nitrite levels in the cultured medium determined by a colorimetric method based on the Griess reagent and sodium nitrite as a standard substance. The cells were pretreated with various concentrations of Pd-Ib (5–20 μg/mL). Two hours later, cells were incubated for another 20 h in the presence of LPS (1 μg/mL) at 37 °C. Then, 100 μL of each supernatant were mixed with an equal volume of Griess reagent. The samples were incubated at room temperature for 15 min. The optical densities were measured at 540 nm by a microplate reader, and nitrite concentration was determined by a standard curve generated with known concentrations of sodium nitrite.

### Statistical analysis

Experiments were performed at least three times as indicated. Data were presented as mean ± SD from three independent experiments. Statistical differences among groups were evaluated by one-way analysis of variance (ANOVA) and Duncan’s multiple range test by IBM SPSS Statistics 20 for Windows (SPSS Inc., Chicago, IL, USA). *P* values <0.05 were considered statistically significant. The dose-dependence was visually determined.

## Results and discussion

### Bioassay-guided isolation and structural identification

As shown in Fig. 1, upon LPS stimulation, The SEAP activity was significantly inhibited by the addition of the PE extract (50 μg/mL) (16.52–9.86 %, *P* < 0.0001). The PE extract was subjected to silica gel chromatography using PE/EtOAc as an eluent and eight fractions were collected to further search for anti-inflammatory compounds. Because the SEAP activity was significantly inhibited by Fr. 3 (24.67–36.65 %, *P* < 0.0001), further purification was carried out to isolate one pure compound. This compound, which inhibited the SEAP activity (IC_50_ = 22.53 μg/mL) in the LPS-stimulated macrophages in a dose-dependent manner, was determined to be Pd-Ib by HR-MS analysis (MicrOToF-Q Bruker mass spectrometer equipped with Acquity Waters ultra-high performance liquid chromatography) and NMR spectra analysis (Bruker 400 Hz NMR spectrometer). The spectral data (Additional files 3 and 4) are identical to those reported [15–17]. To our knowledge, this is the first time that Pd-Ib has been found in *B.malconense.*

Pd-Ib yield: 2.38 mg of white powder (MeOH). HR-ESI^+^-MS: m/z 342.1183 [M+H]^+^ (calculated for C_19_H_18_O_6_, 342.1182). ^1^H-NMR (CDCl_3_, 400 MHz) *δ*: 6.35 (1H, d, *J* = 9. 6 Hz, H-3), 7.63 (1H, d, *J* = 9.6 Hz, H-4), 7.57 (1H, d, *J* = 8.4 Hz, H-5), 6.90 (1H, d, *J* = 8.8 Hz, H-6), 5.69 (1H, s, *J* = 4.8 Hz, H-3′), 1.62 (3H, s, 5′-CH_3_), 1.45 (3H, s, 5′-CH_3_), 6.25 (1H, q, *J* = 7. 5 Hz, H-3″), 2.07 (3H, d, *J* = 8.8 Hz, 4″-CH3), 2.00 (3H, s, 5″-CH_3_).

### Cytotoxicity of Pd-Ib in RAW-Blue cells

**Fig. 2:**
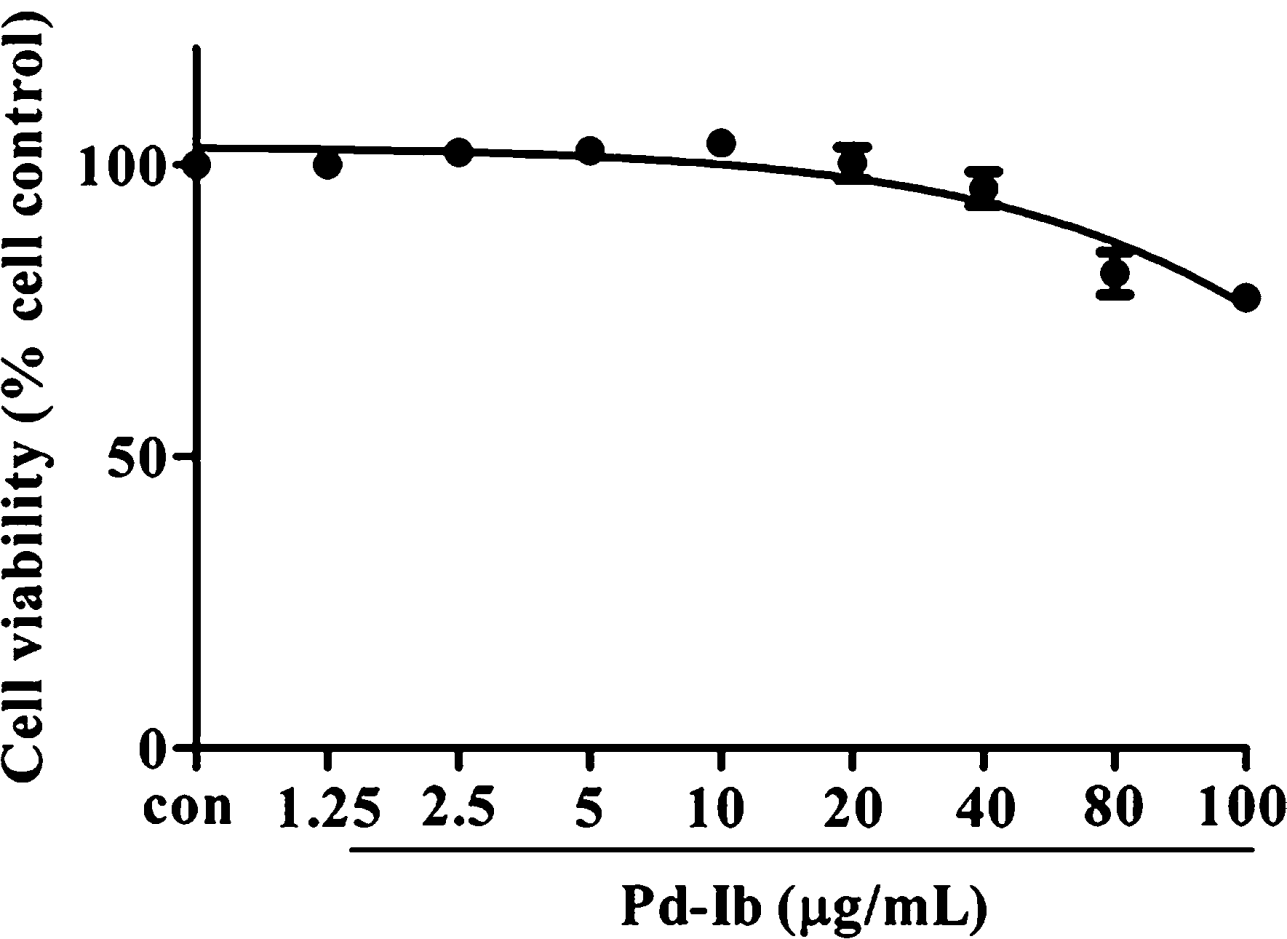
Effect of Pd-Ib on the viability of LPS-stimulated RAW-Blue cells. Cells were cultured for 24 h in the presence of Pd-Ib at the indicated concentrations (1.25–100 μg/mL). Cell viability was assessed by the MTT assay. Data were obtained from three independent experiments and presented as mean ± SD. *con* vehicle control group

The cytotoxic effect of Pd-Ib on RAW-Blue cells was tested with the MTT assay. As shown in Fig. 2, Pd-Ib did not exhibit cytotoxic effects at dosages ranging from 1.56 to 20 μg/mL; however, the cell viability decreased when the macrophages were treated with Pd-Ib at doses of 40, 80 or 100 μg/mL.

### Pd-Ib inhibited the nuclear translocation of NF-κB and decreased degradation of IκB-α in LPS-stimulated RAW-Blue cells

**Fig. 3:**
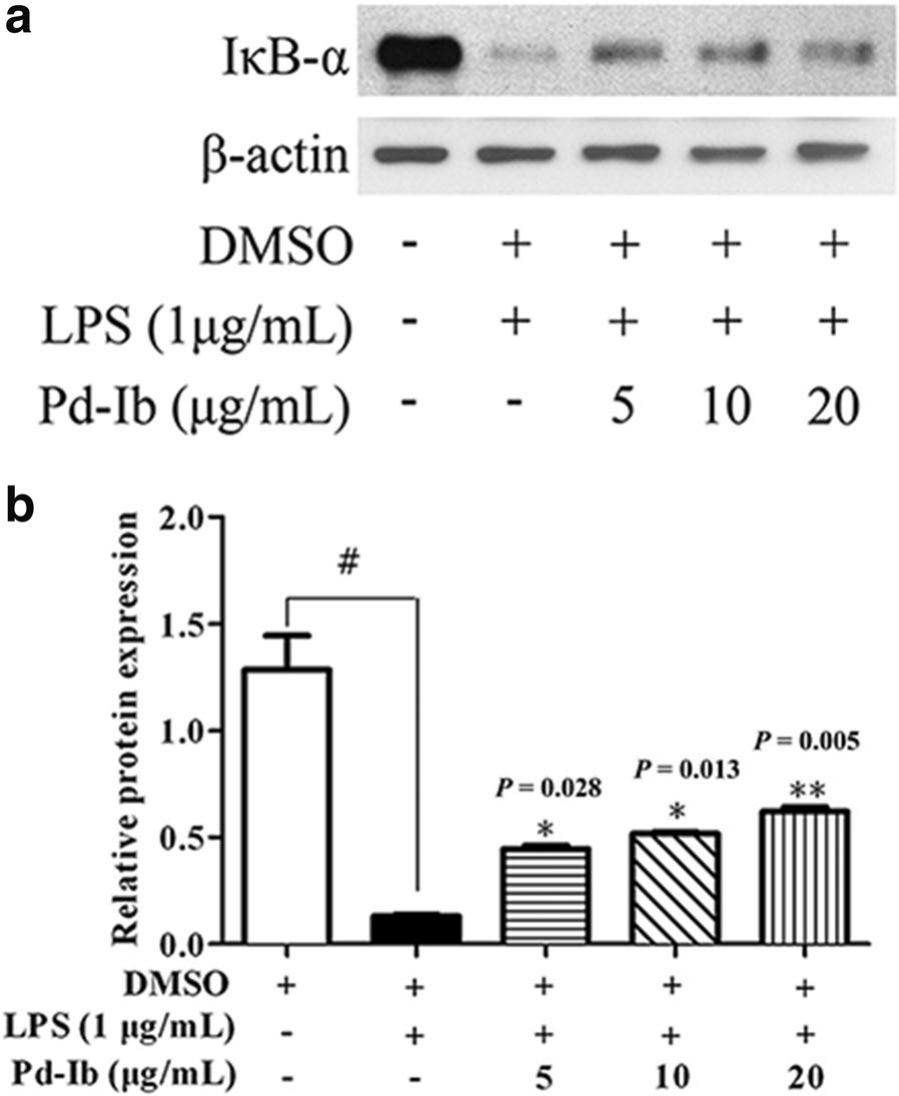
Effect of Pd-Ib on inhibited the IκB-α degradation in LPS-stimulated RAW-Blue cells. After macrophages were treated with 1 μg/mL of LPS in absence or presence of various concentrations of Pd-Ib (5, 10 and 20 μg/mL) for 20 h, the protein level of IκB-α was determined by western blotting, β-actin was used as a loading control. **a** Representative image of western blotting. **b** The protein level of IκB-α was calculated with ImageJ software. Data were derived from three independent experiments and presented as mean ± SD. ^#^ Compared with the control group. *P < 0.05 and **P < 0.01 vs. LPS-alone group

The SEAP activity in the supernatants of the LPS-stimulated RAW-Blue cells reflects the activation of NF-κB [18]. NF-κB activation is involved in a number of cellular processes, particularly inflammation. The inhibition of NF-κB activation was associated with the mitigation of colon inflammation responses and apoptosis of intestinal epithelial cells in the DSS mouse model [19]. In our in vitro study, the immunofluorescence analysis showed that the nuclear translocation of NF-κB p65 subunit in the LPS-stimulated cells was remarkably up-regulated when comparing to that of the non-LPS-treated control, denoting the activation of NF-κB. When Pd-Ib (20 μg/mL) treatment was given, the translocation of NF-κB p65 was inhibited (Fig. 3).

**Fig. 4:**
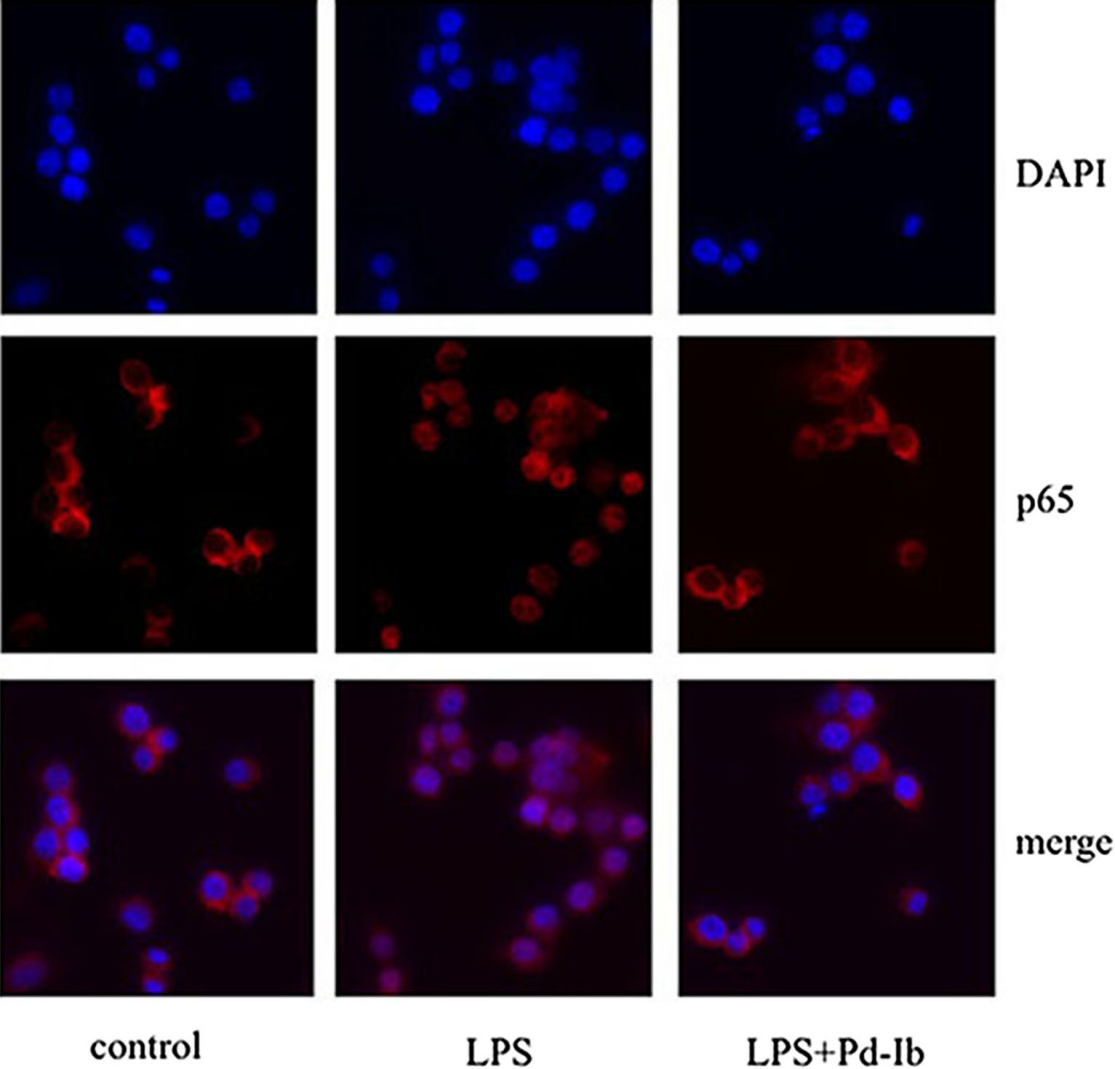
Effect of Pd-Ib on the nuclear translocation of NF-κB. Macrophages were pre-incubated with Pd-Ib (20 μg/mL) for 2 h and then with or without 1 μg/mL of LPS for 20 h. And then Nuclei were stained with DAPI for immunofluorescence staining analysis. Data were derived from three independent experiments. Original magnification, ×40

In contrast, the expression of the inhibitory subunit of NF-κB, IκB-α was clearly depredated in macrophages upon LPS stimulation. From the western blotting images, we demonstrated that the loss of cytoplasmic IκB-α was inhibited by Pd-Ib (5, 10, 20 μg/mL) in a dose-dependent manner when compared with the LPS-stimulated macrophages (*P* = 0.028, *P* = 0.013, *P* = 0.005 for three respective concentrations; Fig. 4).

### Pd-Ib suppressed the expression of iNOS and COX-2 in LPS-stimulated RAW-Blue cells

**Fig. 5:**
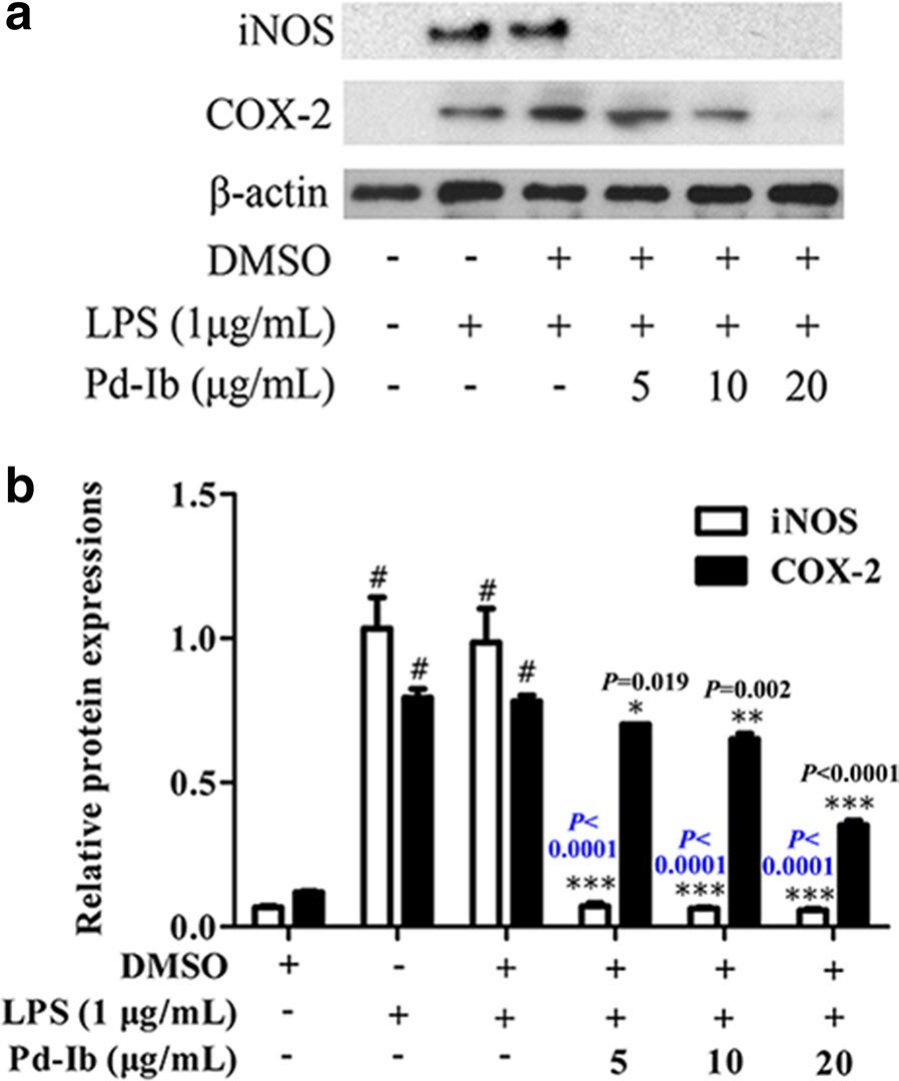
Inhibitory effect of Pd-Ib on iNOS and COX-2 expression in LPS-stimulated RAW-Blue cells. After macrophages were treated with 1 μg/mL LPS in the absence or presence of various concentrations of Pd-Ib (5, 10 and 20 μg/mL) for 20 h, the protein levels of iNOS and COX-2 were determined by western blotting; β-actin was used as a loading control. **a** Representative western blotting images. **b** The protein levels of iNOS and COX-2 were calculated with ImageJ software. Data were derived from three independent experiments and presented as mean ± SD. ^#^Compared with the control group. *P < 0.05, **P < 0.01 and ***P < 0.001 vs. LPS-alone group

COX-2 and iNOS are known to mediate inflammatory responses. High expression levels cause intestinal inflammation with motility dysfunction [20]. Pd-Ib (5, 10, 20 μg/mL) strongly down-regulated iNOS (*P* < 0.0001, *P* < 0.0001, *P* < 0.0001 for three respective concentrations) and COX-2 (*P* = 0.019, *P* = 0.002, *P* < 0.0001 for three respective concentrations) protein levels in a dose-dependent manner (Fig. 5a, b).

**Fig. 6:**
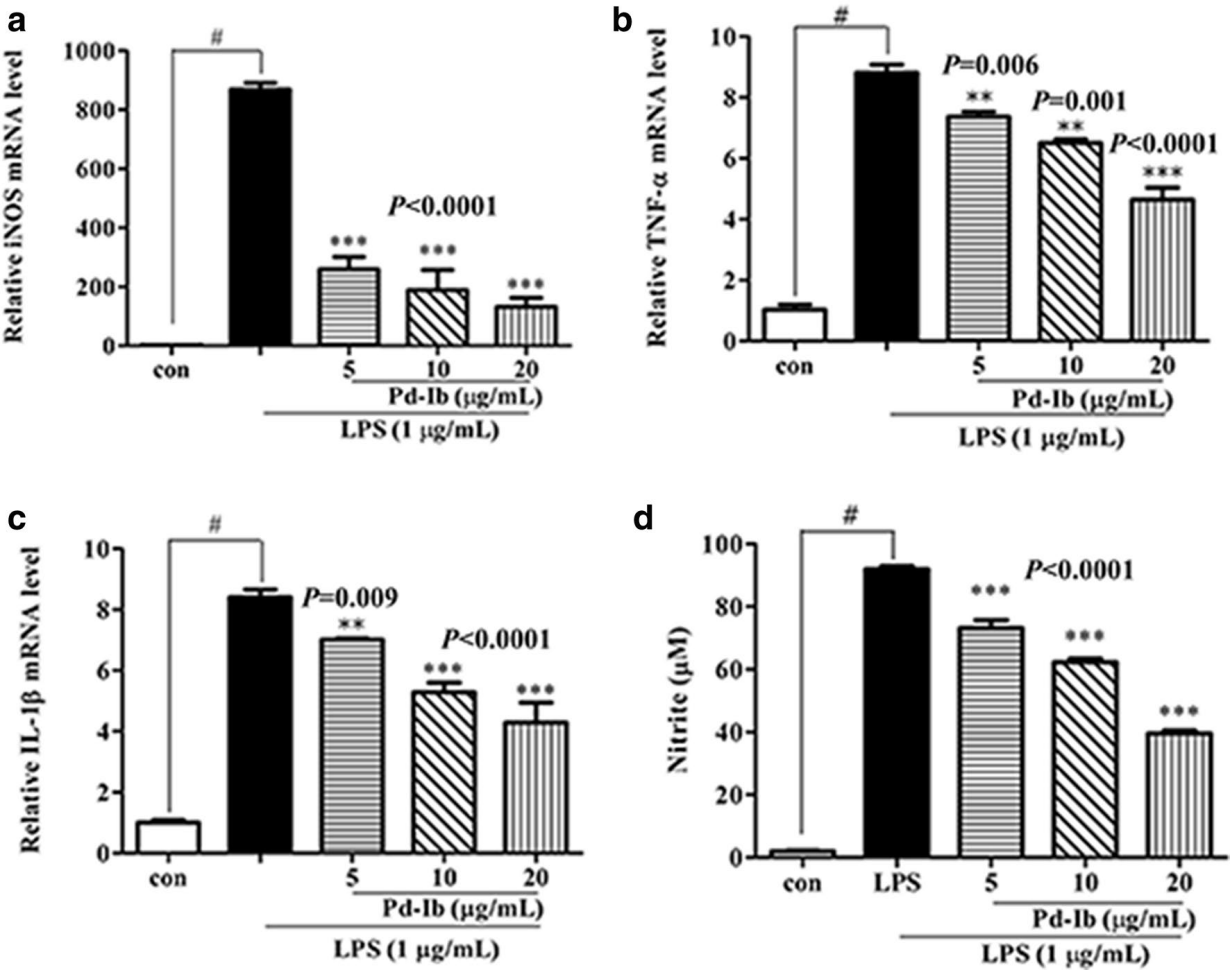
Effect of pro-inflammatory factors expression on nitric oxide production in LPS-stimulated RAW-Blue cells. After macrophages were treated with 1 μg/mL LPS in the absence or presence of various concentrations of Pd-Ib (5, 10 and 20 μg/mL) for 20 h, the mRNA levels of *iNos* (**a**) *Tnf*-*α* (**b**) and *Il*-*1β* (**c**) were determined by real-time PCR, and the production of NO (**d**) was determined by Griess reagent. Data were derived from three independent experiments and presented as mean ± SD. *con* vehicle control group. ^#^Compared with the control group. *P < 0.05, **P < 0.01 and ***P < 0.001 vs. LPS-alone group

Furthermore, the effect of Pd-Ib on the mRNA expression of *iNos* was investigated in LPS-stimulated RAW-Blue cells. As shown in Fig. 6a, Pd-Ib (5, 10, 20 μg/mL) also markedly inhibited the mRNA level of *iNos* (*P* < 0.0001, *P* < 0.0001, *P* < 0.0001 for three respective concentrations) in a dose-dependent manner. Pd-Ib plays a key role in the inhibition of iNOS protein and gene expression.

### Pd-Ib inhibited the mRNA expression of *Tnf*-*α* and *Il*-*1β* in LPS-stimulated RAW-Blue cells

High levels of pro-inflammatory cytokines are associated with pain, lung inflammation and rheumatoid arthritis [21–23]. In this study, the mRNA expression of pro-inflammatory cytokines was examined by real-time qPCR. As shown in Fig. 6b, c, Pd-Ib (5, 10, 20 μg/mL) inhibited the mRNA expression of *Tnf*-*α* (*P* < 0.006, *P* < 0.001, *P* < 0.0001 for three respective concentrations) and *Il*-*1β* (*P* < 0.001, *P* < 0.0001, *P* < 0.0001 for three respective concentrations) in a dose-dependent manner, while LPS stimulation of macrophages caused an increase in their expression.

### Pd-Ib inhibited NO production in LPS-stimulated RAW-Blue cells

Production of excessive amounts of NO leads to inflammatory responses or tissue injury [24]. As shown in Fig. 6d, LPS stimulation significantly increased NO production compared with the control group, while treatment with 5, 10 or 20 μg/mL Pd-Ib led to 17.64 ± 2.96, 29.82 ± 1.34 and 55.45 ± 1.15 % (*P* < 0.0001, *P* < 0.0001, *P* < 0.0001 for three respective concentrations) inhibition of NO production, respectively.

Pd-Ib exerted anti-inflammatory effects through inhibition of SEAP activity in the QUANTI-Blue assay. SEAP is a reporter gene widely used to screen immune-pharmacological activities upon the activation of NF-κB and activator protein 1 (AP-1) [25, 26]. When RAW-Blue cells are stimulated with LPS, IκB-α, which is the inhibitory subunit of NF-κB, is rapidly phosphorylated. Subsequently, NF-κB releases from the IκB-α subunit and translocates to the nucleus, where it increases the expression of the genes that encode many cytokines, enzymes and adhesion molecules [27]. Pd-Ib significantly inhibited the LPS-stimulated degradation of IκB-α and the nuclear translocation of NF-κB.

Increased expression of two key factors regulated by NF-κB, COX-2 and iNOS, is reflected in the increased amount of NO in the colon of patients with active ulcerative colitis [28]. Pd-Ib significantly inhibited the protein expression of COX-2 and iNOS, resulting in a decrease in NO in activated macrophages. The results also suggest that Pd-Ib down-regulated the protein levels of iNOS by reducing its mRNA expression.

When the pro-inflammatory cytokines, including TNF-α and IL-1β, are overproduced, often lead to inflammatory process [29]. Thus, inhibition of the release of pro-inflammatory cytokines may attenuate the inflammatory response [30]. Our qPCR results showed that Pd-Ib significantly down-regulated the mRNA expression of *Tnf*-*α* and *Il*-*1β* in a dose-dependent manner. Our finding of Pd-Ib agreed to the previous studies [31–33] that many natural compounds inhibiting TNF-α activation suppress the nuclear expression of NF-κB.

## Conclusion

Pd-Ib isolated from *B.malconense* suppressed LPS-induced inflammatory responses in macrophages by inhibiting NF-κB activity and reducing the expression of iNOS, COX-2 as well as pro-inflammatory cytokines.

## Additional files

10.1186/s13020-016-0077-x Effects of PE extract of *B. malconese* on body weight change (A) and colon length (B). Colitis was induced in all groups except the control group. PE extract administered to mice for seven days. The change in body weight was taken as the difference between the body weight before induction of colitis and that immediately before sacrifice on day 7. On day 7, the mice were sacrificed, and the colon lengths were measured. Data are expressed as mean ± SD, n = 5 (^#^ *P* < 0.001, compared with the control group; **P* < 0.05, compared with DSS model group).
10.1186/s13020-016-0077-x NJ tree constructed by MEGA 4.0 based on *psb*A-*trn*H of 19 taxa of *Dendrobium* and one inspected species.
10.1186/s13020-016-0077-x HR-ESI-MS spectrum of Pd-Ib.
10.1186/s13020-016-0077-x ^1^H NMR spectrum of Pd-Ib.

## Abbreviations

LPS: lipopolysaccharide
Pd-Ib: (+)-3′-angeloxyloxy-4′-keto-3′,4′-dihydroseselin
qPCR: quantitative polymerase chain reaction
IL-1β: interleukin 1 beta
TNF-α: tumor necrosis factor-alpha
iNOS: inducible nitric oxide synthase
COX-2: cyclooxygenase-2
NO: nitric oxide
NF-κB: nuclear factor kappa-light-chain-enhancer of activated B cells
IκB-α: nuclear factor of kappa light polypeptide gene enhancer in B-cells inhibitor, alpha
DSS: dextran sulfate sodium
MTT: 3-[4,5-dimethylthiazol-2-yl]-2,5-diphenyltetrazolium bromide
DMSO: dimethyl sulfoxide
DMEM: Dulbecco’s modified Eagle’s medium
FBS: fetal bovine serum
PE: petroleum ether
EtOAc: ethyl acetate
Fr: fraction
EtOH: ethanol extract
H_2_O: water extract
con: vehicle control group
SEAP: secreted embryonic alkaline phosphatase
SDS-PAGE: sodium dodecylsulphate-polyacrylamide gel
TBST: tris-buffered saline-Tween
BSA: bovine serum albumin
PBS: phosphate buffer saline
DAPI: 4′,6-diamidino-2-phenylindole
AP-1: activator protein 1
