# Supplementary figures and images for: A novel coumarin, (+)-3′-angeloxyloxy-4′-keto-3′,4′-dihydroseselin, isolated from Bupleurummalconense (Chaihu) inhibited NF-κB activity
Source: Chin Med. 2016 Feb 13;11:5. doi: 10.1186/s13020-016-0077-x (PMC4752810; doi:10.1186/s13020-016-0077-x)

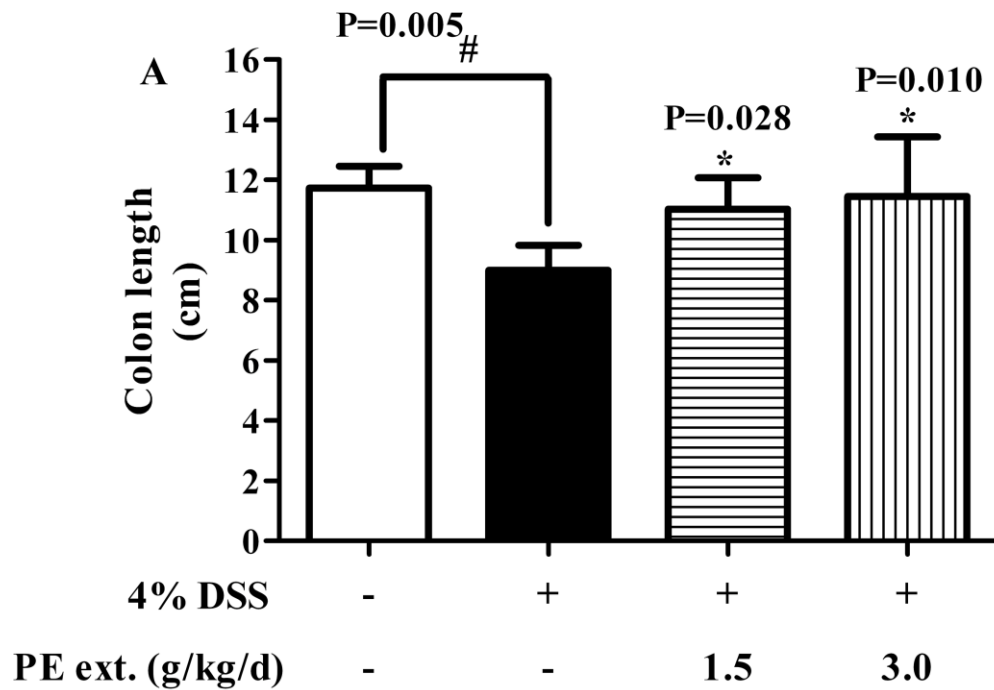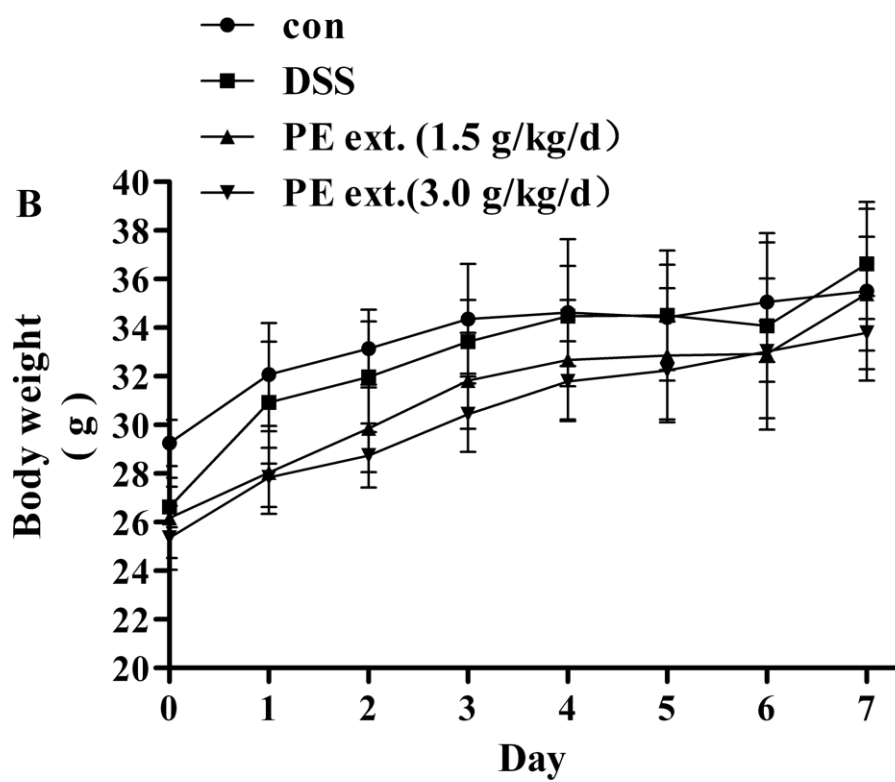

Supplement: Supplementary file 1 — 10.1186/s13020-016-0077-x Effects of PE extract of B. malconese on body weight change (A) and colon length (B). Colitis was induced in all groups except the control group. PE extract administered to mice for seven days. The change in body weight was taken as the difference between the body weight before induction of colitis and that immediately before sacrifice on day 7. On day 7, the mice were sacrificed, and the colon lengths were measured. Data are expressed as mean ± SD, n = 5 (# P < 0.001, compared with the control group; *P < 0.05, compared with DSS model group). [file 13020_2016_77_MOESM1_ESM.pdf]

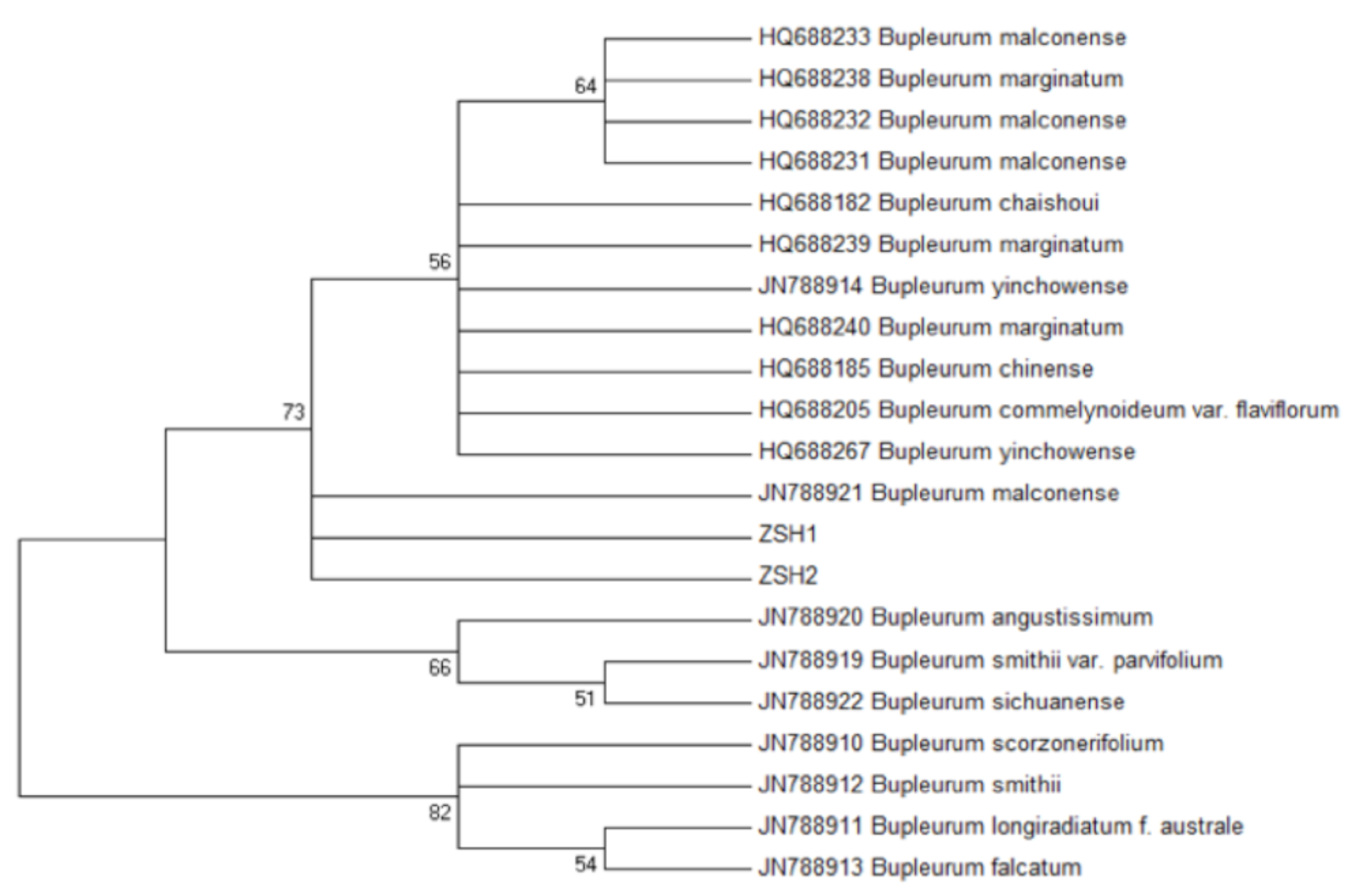

Supplement: Supplementary file 2 — 10.1186/s13020-016-0077-x NJ tree constructed by MEGA 4.0 based on psbA-trnH of 19 taxa of Dendrobium and one inspected species. [file 13020_2016_77_MOESM2_ESM.tiff]

# Window Display Report

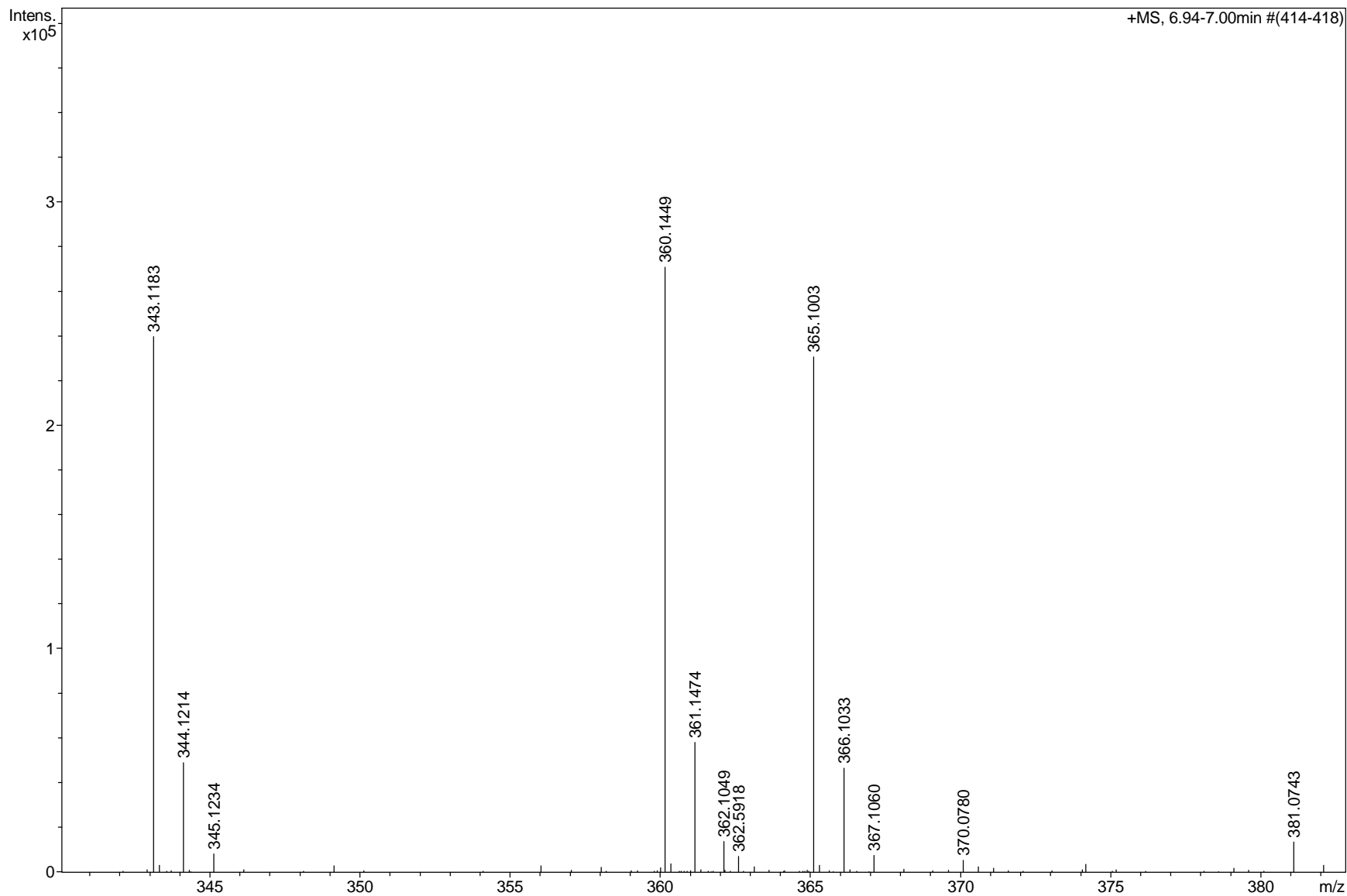

Supplement: Supplementary file 3 — 10.1186/s13020-016-0077-x HR-ESI-MS spectrum of Pd-Ib. [file 13020_2016_77_MOESM3_ESM.pdf]

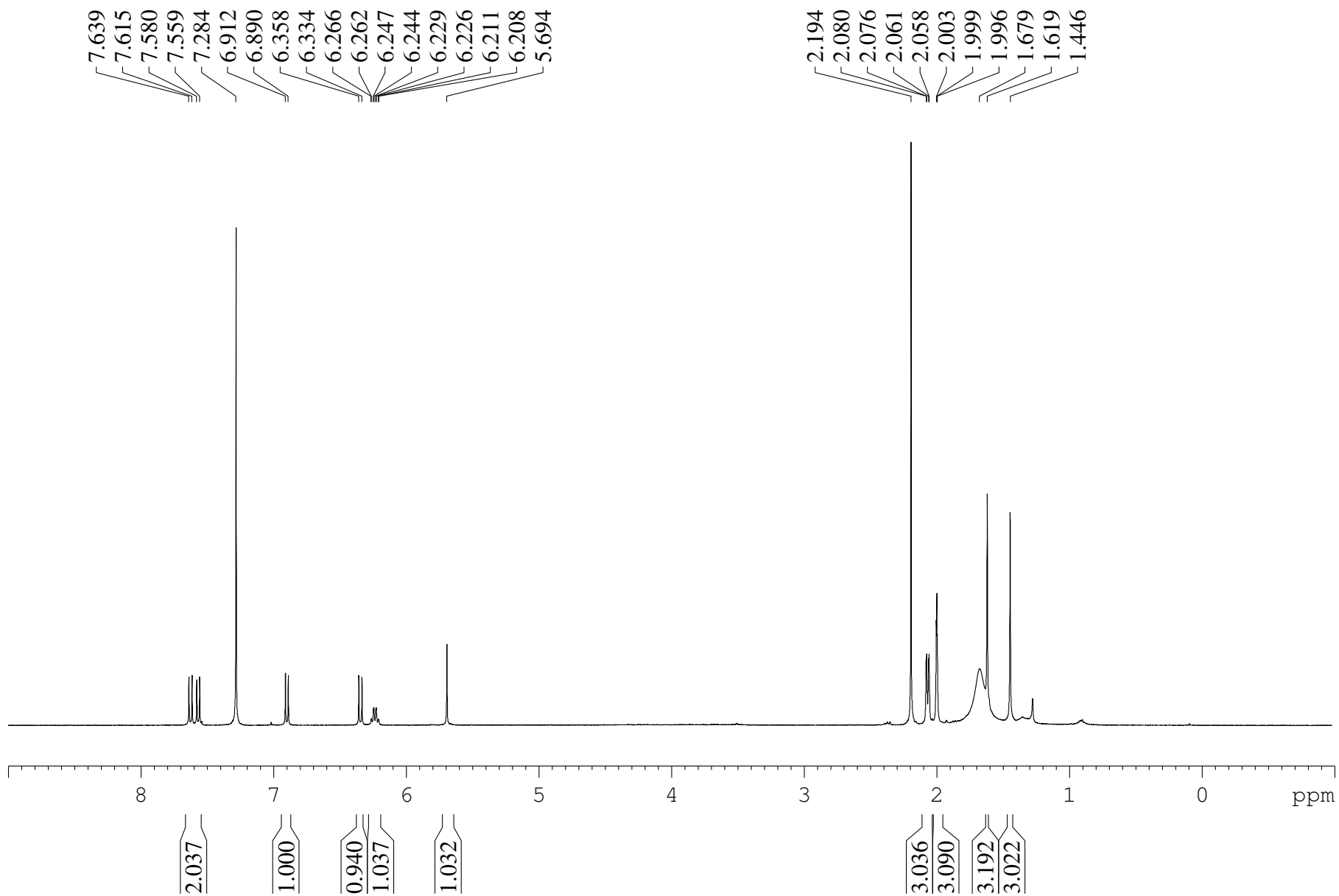

Supplement: Supplementary file 4 — 10.1186/s13020-016-0077-x 1H NMR spectrum of Pd-Ib. [file 13020_2016_77_MOESM4_ESM.pdf]
